# Establishment of Specific Multiplex PCR Detection Methods for the Predominant *tet*(X)-Positive *Acinetobacter* Species

**DOI:** 10.3390/microorganisms13112584

**Published:** 2025-11-12

**Authors:** Chong Chen, Jing Liu, Jie Gao, Taotao Wu, Jinlin Huang

**Affiliations:** 1 Joint International Research Laboratory of Agriculture and Agri-Product Safety, Ministry of Education of China, Institutes of Agricultural Science and Technology Development, Yangzhou University, Yangzhou 225009, China; chen_chong@yzu.edu.cn (C.C.);; 2 College of Bioscience and Biotechnology, Yangzhou University, Yangzhou 225009, China

**Keywords:** *A. indicus*, *A. amyesii*, *A. towneri*, *tet*(X), multiplex PCR detection

## Abstract

The increasing prevalence of the mobile tigecycline resistance gene *tet*(X) poses a severe global health threat, and the genus *Acinetobacter* is a major reservoir. This study aimed to develop a rapid and specific multiplex PCR assay for detecting the predominant *tet*(X)-positive *Acinetobacter* species. Through pan-genome analyses of 390 *tet*(X)-positive *Acinetobacter* genomes, a total of 20 *tet*(X) variants were identified in 24 *Acinetobacter* species, including 17 published lineages and seven taxonomically unresolved Taxa. *Acinetobacter indicus* (30.8%), *Acinetobacter amyesii* (17.2%), and *Acinetobacter towneri* (16.1%) were the top three hosts of diverse *tet*(X) variants. Species-specific signature genes were identified and used for primer design, yielding amplicons of 267 bp (*tet*(X)), 424 bp (*A. indicus*), 690 bp (*A. amyesii*), and 990 bp (*A. towneri*). The assay was rigorously adjusted for an optimal annealing temperature of 52.8 °C and a primer ratio of 1:1:1:1, demonstrating high sensitivity with a detection limit of 0.3 ng/μL DNA and excellent stability under −20 °C, 4 °C, 20 °C storage conditions. Validation experiments on 151 bacterial strains showed high accuracy for DNA templates (≥97.8%) and bacterial suspensions (≥93.5%) within two hours. This cost-effective and highly accurate multiplex PCR provides a powerful tool for proactive surveillance and control of the critical *Acinetobacter* sp. pathogens.

## 1. Introduction

Among clinically important pathogens, *Acinetobacter* sp. isolates have drawn considerable attention due to their strong pathogenicity, adaptability, and adhesive capacity. Bacteria of this genus can survive on both biological and non-biological surfaces, and represent causative agents of ventilator-associated pneumonia, bloodstream infections, and surgical site infections [1,2]. The genomic DNA is prone to mutation and recombination, leading to 90 officially reported species and more than 70 unknown species to be named (https://lpsn.dsmz.de/genus/acinetobacter (accessed on 6 November 2025)) [3]. *Acinetobacter baumannii* is one of the most important pathogenic bacteria in the clinical setting, followed by *Acinetobacter calcoaceticus*, *Acinetobacter pittii*, *Acinetobacter junii*, *Acinetobacter lwoffii*, *Acinetobacter nosocomialis*, and *Acinetobacter seifertii* [4,5,6,7,8,9]. With the widespread use of antibiotics, the problem of antimicrobial resistance in *Acinetobacter* spp. has become increasingly prominent. In 2019, the median proportion of patients with bloodstream infections caused by carbapenem-resistant *Acinetobacter* spp. was 70.3% in the Eastern Mediterranean Region, and the infections from carbapenem-resistant *Acinetobacter baumannii* (CRAb) were responsible for global 57,770 deaths [9,10]. Highlighting its grave threat, the World Health Organization classified CRAb as a critical-priority pathogen on its 2024 Bacterial Priority Pathogens List [11].

Tigecycline, a third-generation tetracycline antibiotic derived from the structural basis of minocycline, exhibits broad-spectrum antimicrobial activities against Gram-negative and Gram-positive bacteria by inhibiting protein synthesis [12]. Subsequently, a mobile tigecycline resistance gene *tet*(X3) was first reported in porcine *A. baumannii* in 2019 in China [13]. This gene conferred a broader resistance profile than the previously identified *tet*(X) (GenBank accession number: M37699) and *tet*(X2) (AJ311171), even posing a threat to the latest generation of tetracycline antibiotics such as eravacycline and omadacycline [14]. To date, the *tet*(X2), *tet*(X3), *tet*(X4), *tet*(X5), *tet*(X6), *tet*(X7), *tet*(X13), and *tet*(X15) variants have been identified in multiple *Acinetobacter* species, especially in China [1]. They were particularly prevalent in carbapenemase NDM-producing strains such as *A. baumannii*, *A. indicus*, *A. lwoffii*, *Acinetobacter schindleri*, *A. towneri*, and *Acinetobacter johnsonii*, further exacerbating the challenges in clinical treatment [13,15,16,17].

Now there is an urgent need in the fields of clinical microbiology and public health to develop rapid and accurate detection technologies for multidrug-resistant (MDR) pathogens. Among these, the PCR technology has become one of the most powerful tools in pathogenic microbe detection due to its high sensitivity and specificity. Conventional PCR can identify a variety of bacterial pathogens, such as *Acinetobacter* spp., *Escherichia coli*, *Klebsiella pneumoniae*, and *Salmonella enterica*, by 16S rRNA sequencing and alignment [18,19]. Multiplex PCR, developed based on conventional PCR, further enhances detection efficiency. For example, five pairs of primers were designed to establish a multiplex PCR method capable of simultaneously detecting *Vibrio alginolyticus*, *Vibrio parahaemolyticus*, *Vibrio vulnificus*, and *Vibrio cholerae* [20]. The other techniques, such as biochemical experiments, quantitative real-time PCR (qPCR), whole genome-based Average Nucleotide Identity (ANI), microfluidic chips, and matrix-assisted laser desorption/ionization time-of-flight mass spectrometry (MALDI-TOF MS), also play important roles in pathogenic identification [21,22,23,24,25]. Despite continuous advancements in existing technologies, there remains a lack of specific, rapid, and cost-effective methods for detecting *tet*(X)-positive *Acinetobacter* sp. strains. To solve the limitation, a multiplex PCR amplification system targeting three predominant *tet*(X)-positive *Acinetobacter* species was explored in this study.

## 2. Materials and Methods

### 2.1. Genome Collection and Quality Evaluation

With the complete nucleotide sequence of *tet*(X3) (MK134375) as a query template, all non-duplicate *tet*(X)-carrying *Acinetobacter* genomes and related bacterial information were retrieved from the public National Center for Biotechnology Information (NCBI) database (accessed on 5 January 2025). Then they were subject to quality evaluation by QUAST version 5.2.0 and CheckM version 1.1.6 for the next analyses [26,27]. The filter parameters included the number of contigs (<300), N50 (>40 kb), completeness (>95%), contamination (<2%), and heterogeneity (≤50%).

### 2.2. Identification of Acinetobacter Species, Antibiotic Resistance Genes, and Sequence Types (STs)

An ANI analysis was conducted for precise bacterial identification of *Acinetobacter* species. ANI values between each of the qualified *tet*(X)-positive genomes and type strains of *Acinetobacter* spp. were calculated using FastANI version 1.33, and a cutoff of >95% was used to define the same bacterial species [24]. The *tet*(X) subtypes were manually analyzed by multiple sequence alignment with previously reported variants, with the threshold values of 100% amino acid identity and 100% amino acid coverage [28]. The other antibiotic resistance genes were detected in the Comprehensive Antibiotic Resistance Database (CARD) using ABRicate version 1.0.1, with the threshold values of >98% nucleotide identity and >98% nucleotide coverage (https://github.com/tseemann/abricate (accessed on 6 November 2025)). STs were analyzed against the PubMLST abaumannii_2 scheme by MLST version 2.22.0, with the threshold values of 100% nucleotide identity and 100% nucleotide coverage (https://github.com/tseemann/mlst (accessed on 6 November 2025)).

### 2.3. Primer Design Based on Pan-Genome Analyses

Pan-genomes of three predominant *tet*(X)-positive *Acinetobacter* species were analyzed under default parameters by IPGA version 1.09, respectively [29]. Core gene clusters were selected for online species-specific primer design by the section of Primers common for a group of sequences in Primer-BLAST using Primer3 version 2.5.0, respectively, with melting temperatures ranging from 48 °C to 60 °C in the nt database (https://blast.ncbi.nlm.nih.gov/Blast.cgi (accessed on 16 May 2024)). Similarly, a pair of primers for *tet*(X) variants in *Acinetobacter* sp. strains was also designed. Particularly, the above primers were all designed to generate PCR products of significantly different sizes for bacterial detection (Table 1).

### 2.4. Primer Confirmation

To verify the primers, bacterial genomes of *tet*(X)-positive *A. indicus* C20230218, *A. amyesii* YH16040, and *A. towneri* TT6-2 were extracted using a TIANamp Bacteria DNA Kit (Tiangen, Beijing, China). Genomic DNA was then adjusted to a concentration of 30 ng/μL, respectively, of which 1 μL was used as a template for PCR amplification, agarose gel electrophoresis, Sanger sequencing, and sequence alignment. The PCR reaction system and procedure using 2 × Taq Master Mix (Vazyme, Nanjing, China) were shown in Tables S1 and S2, and *A. baumannii* ATCC 19606 was used as the negative control group.

### 2.5. Determination of the Optimal Annealing Temperature

Genomic DNA from *tet*(X)-positive *A. indicus* C20230218, *A. amyesii* YH16040, and *A. towneri* TT6-2 were mixed in equal proportions at the same concentration and used as the PCR template. PCR amplification was performed using an equimolar primer mixture at different annealing temperatures of 47.0 °C, 48.5 °C, 49.9 °C, 51.4 °C, 52.8 °C, 54.3 °C, 55.7 °C, 57.2 °C, 58.6 °C, 60.1 °C, 61.5 °C, and 63.0 °C, with three replicates per group, to determine the optimal annealing temperature for the multiplex PCR system. Additionally, ImageJ version 1.8.0 was used to analyze the grayscale intensity of DNA bands.

### 2.6. Determination of the Optimal Primer Ratio

Four pairs of primers tetX-F/R, indicus-F/R, amyesii-F/R, and towneri-F/R (each at a concentration of 10 μM) were mixed in different ratios of 1:1:1:1, 1:2:1:1, 1:1:2:1, 1:1:1:2, or 1:1:1:3 with a total volume of 4 μL. Using mixed genomic DNA from three *tet*(X)-positive *Acinetobacter* sp. strains as the PCR template, PCR amplification was performed under the optimal annealing temperature, with three replicates for each group, to determine the optimal primer ratio for the multiplex PCR system. Additionally, ImageJ version 1.8.0 was used to analyze the grayscale intensity of DNA bands.

### 2.7. Determination of the Minimum Detection Limit

Sterile water was used to prepare a two-fold serial dilution of the mixed genomic DNA extracted from three *tet*(X)-positive *Acinetobacter* sp. strains, which served as the PCR template. Subsequently, PCR amplification was performed under the optimal primer ratio and optimal annealing temperature, with three replicates per group, to determine the limit of detection of the multiplex PCR system.

### 2.8. Primer Stability Under Different Storage Temperatures

The specific primers were mixed at the optimal ratio and aliquoted into nine tubes. These aliquots were stored under three temperature conditions of −20 °C, 4 °C, and 20 °C, with three biological replicates per group, to evaluate the stability of multiplex PCR primers targeting *tet*(X)-positive *Acinetobacter* species. PCR amplification was performed at the predetermined optimal annealing temperature at five time points of day 1, day 3, day 7, day 10, and day 15. Simultaneously, DNA band intensity was quantitatively analyzed using ImageJ version 1.8.0 for the grayscale measurement.

### 2.9. Determination of the Multiplex PCR Detection Accuracy

A total of 151 bacterial strains preserved in our laboratory were tested for the accuracy of the multiplex PCR method, consisting of 145 *tet*(X)-positive strains and six *tet*(X)-negative control strains. The *tet*(X)-positive strains contained *A. indicus* (*n* = 46), *A. amyesii* (*n* = 17), *A. towneri* (*n* = 8), *Acinetobacter variabilis* (*n* = 28), *A. schindleri* (*n* = 5), *Acinetobacter pseudolwoffii* (*n* = 5), *Acinetobacter sichuanensis* (*n* = 2), *A. lwoffii* (*n* = 1), *Acinetobacter defluvii* (*n* = 1), *E. coli* (*n* = 9), *Aeromonas caviae* (*n* = 1), *Empedobacter stercoris* (*n* = 13), *Myroides tengzhouensis* (*n* = 1), *Myroides odoratimimus* (*n* = 5), *Myroides zaozhuangensis* (*n* = 1), and *Myroides faecalis* (*n* = 2). In addition, the *tet*(X)-negative control strains included *E. coli* C600, *E. coli* ATCC 25922, *A. baumannii* ATCC 19606, *Acinetobacter baylyi* ADP1, *S. enterica* ATCC 14028, and *K. pneumoniae* ATCC 700603. PCR amplification was performed using their genomic DNA as templates at the optimal annealing temperature and optimal primer ratio. Meanwhile, the bacterial suspensions before genome extraction were also detected under the same conditions.

## 3. Results

### 3.1. Distribution of tet(X) and Associated Genes in Acinetobacter Species

**Figure 1:**
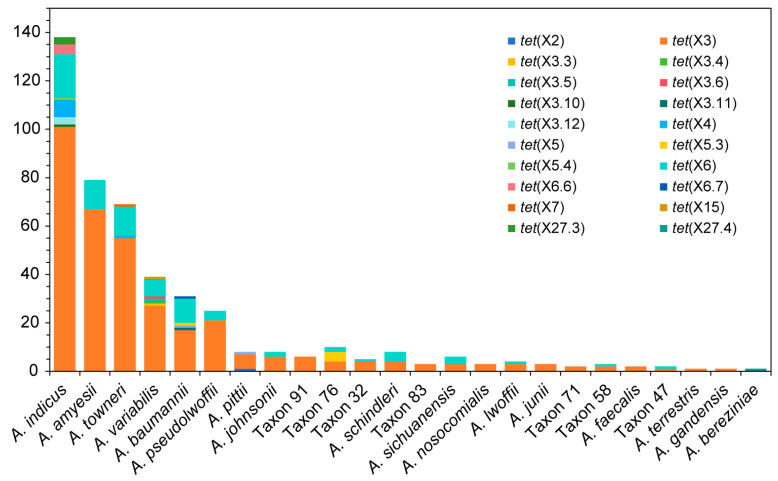
Distribution of the *tet*(X) variants in *Acinetobacter* species. Bacterial information (*n* = 390) is detailed in Table S3.

According to the query and evaluation, 390 *tet*(X)-positive *Acinetobacter* genomes were collected in the NCBI database, of which 63 strains harbored two or more *tet*(X) variants (Table S3). Then the precise distribution of *tet*(X) variants on *Acinetobacter* genomes in China (*n* = 351), USA, (*n* = 7), Pakistan (*n* = 6), Czech Republic (*n* = 4), Thailand (*n* = 4), Germany (*n* = 3), Canada (*n* = 2), Colombia (*n* = 2), Ghana (*n* = 2), Ireland (*n* = 2), Netherlands (*n* = 2), Argentina (*n* = 1), Israel (*n* = 1), Peru (*n* = 1), Philippines (*n* = 1), and Viet Nam (*n* = 1) was systematically delineated. A total of 20 *tet*(X) variants were identified in 24 ANI-based *Acinetobacter* species, including 17 published lineages and seven taxonomically unresolved Taxa (Figure 1). The variants exhibited pronounced inter-species heterogeneity, and *A. indicus* (30.8%), *A. amyesii* (17.2%), and *A. towneri* (16.1%) emerged as the principal hosts, accounting for 64.1% of all *tet*(X)-positive isolates. For *A. indicus*, the main *tet*(X) variant carried by these strains was *tet*(X3) (84.2%), while *tet*(X6) (15%), *tet*(X3.10) (0.8%), *tet*(X3.12) (2.5%), *tet*(X4) (5.8%), *tet*(X5.4) (0.8%), *tet*(X6.4) (3.3%), and *tet*(X27.3) (2.5%) were sporadically detected. For *A. amyesii*, the main *tet*(X) variant was *tet*(X3) (100%), followed by *tet*(X6) (17.9%). For *A. towneri*, *tet*(X3) (87.3%) was the main variant, followed by *tet*(X6) (19%), while *tet*(X4) and *tet*(X7) were sporadically detected (1.6% each). For all the other 21 species, *tet*(X3) was also dominant (85%), followed by *tet*(X6) (25.7%), *tet*(X2) (0.7%), *tet*(X3.3) (0.7%), *tet*(X3.4) (0.7%), *tet*(X3.5) (0.7%), *tet*(X3.6) (0.7%), *tet*(X3.11) (0.7%), *tet*(X5) (1.4%), *tet*(X5.3) (3.6%), *tet*(X6.5) (0.7%), *tet*(X15) (0.7%), and *tet*(X27.4) (0.7%).

As shown in Figure S1, 21 different antibiotic resistance genes, belonging to aminoglycosides, carbapenems, lincosamides, macrolides, phenicols, polypeptides, sulfonamides, and tetracyclines, were analyzed in 390 *tet*(X)-positive *Acinetobacter* sp. bacteria. The detection rate of *sul2* was the highest (94.9%), followed by *floR* (62.8%), *mph*(E) (60%), *aph(6)-Id* (58.7%), *aph(3″)-Ib* (58.5%), *msr*(E) (55.1%), *aac(3)-IId* (26.2%), and *tet*(39) (21%), and those of the remaining genes were <20%. It is noted that the detection rates of carbapenem resistance genes *bla*_OXA-58_, *bla*_NDM-1_, and *bla*_NDM-3_ were 15.1%, 11.3%, and 0.5%, respectively. Results of the ST analysis revealed 85 out of 390 strains were successfully typed, consisting of 39 different STs (Table S3). ST2012 was the most prevalent type (*n* = 13) of them, with <10 for each of the other STs, and the unclassified *Acinetobacter* sp. strains urgently needed further research.

### 3.2. Optimized Primers of Three Predominan tet(X)-Positive Acinetobacter Species

**Figure 2:**
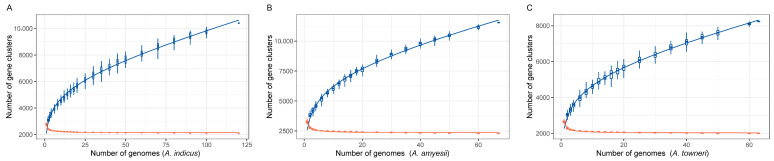
Pan-genome analyses of three predominant *tet*(X)-positive *Acinetobacter* species. Core- and pan-genome profiles of *A. indicus* (**A**), *A. amyesii* (**B**), and *A. towneri* (**C**) are presented in red and blue colors, respectively.

Strains of *A. indicus*, *A. amyesii*, and *A. towneri*, the three most prevalent *tet*(X)-positive species in the NCBI database, were selected as target organisms. By contrast, PPanGGOLiN was the best analytical model in this study. *A. amyesii* exhibited a higher genomic diversity (pan-gene clusters, *n* = 11,558; core-gene clusters, *n* = 2288) than those of *A. indicus* (*n* = 10,403; *n* = 2083) and *A. towneri* (*n* = 8238; *n* = 1995; Figure 2). Conserved signature genes of each species were extracted for *tet*(X)-positive *Acinetobacter* species-specific primers. Consequently, the core gene clusters of *tet*(X) in all strains, thioesterase-coding *acyl-CoA* in *A. indicus*, Major Facilitator Superfamily transporter gene in *A. amyesii*, and flagellar-encoding *filF* in *A. towneri* were successfully applied for primer design by NCBI Primer-BLAST, with the theoretical products of 267 bp, 424 bp, 690 bp, and 990 bp, respectively (Table 1).

**Figure 3:**
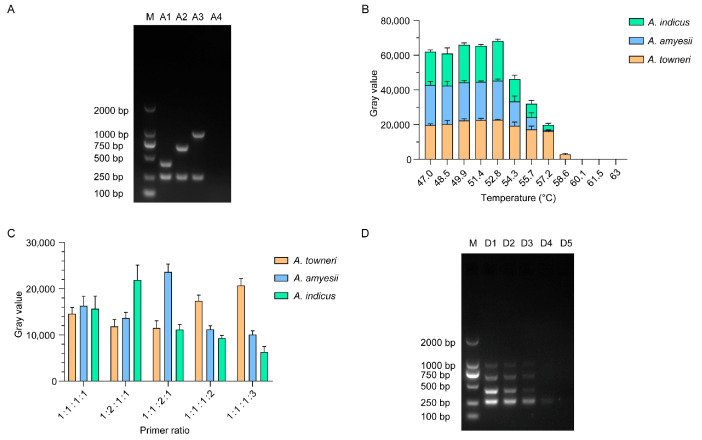
Validation and optimization of PCR primers. Single species-specific PCR detection (**A**), multiplex PCR detection gray values under different annealing temperatures (**B**), multiplex PCR detection gray values under different primer combinations (**C**), and multiplex PCR detection accuracy (**D**) are presented, respectively. Four pairs of primers tetX-F/R, indicus-F/R, amyesii-F/R, and towneri-F/R are mixed in different ratios of 1:1:1:1, 1:2:1:1, 1:1:2:1, 1:1:1:2, and 1:1:1:3. M, DL 2000 DNA Marker; A1, *tet*(X)-positive *A. indicus*; A2, *tet*(X)-positive *A. amyesii*; A3, *tet*(X)-positive *A. towneri*; A4, *tet*(X)-negative control (*A. baumannii* ATCC 19606); D1, 10^−0^ dilution; D2, 10^−1^ dilution; D3, 10^−2^ dilution; D4, 10^−3^ dilution; D5, blank control (without genomic DNA).

PCR and agarose gel electrophoresis results showed clear and expected bands for the representative strains, including *tet*(X)-positive *A. indicus* C20230218, *A. amyesii* YH16040, and *A. towneri* TT6-2 (Figure 3A). They were confirmed by Sanger sequencing and sequence alignment. A multiplex PCR regime was further optimized with respect to the annealing temperature and primer ratio. As illustrated in Figure 3B, amplicons corresponding to each *Acinetobacter* species were obtained across the 47.0–57.2 °C range; however, maximal band sharpness was achieved at 52.8 °C, with the grayscale intensity of 22,950.4 ± 884.7 (*A. indicus*), 22,536.6 ± 838.7 (*A. amyesii*), and 22,761.7 ± 283.9 (*A. towneri*), respectively. Elevating the temperature to ≥58.6 °C resulted in complete loss of amplification for *A. indicus* and *A. amyesii*, whereas the *A. towneri* signal vanished at ≥60.1 °C. Consequently, 52.8 °C was adopted as the optimal annealing temperature for the multiplex PCR assay. To optimize the primer ratio, equimolar mixtures of genomic DNA from the three target species were amplified at the previously determined optimal annealing temperature of 52.8 °C. Densitometric quantification of the resulting amplicons (Figure 3C) revealed that a 1:1:1:1 ratio of four primer pairs produced the smallest inter-band variation in gray-level intensity (15,691.7 ± 2231.4, *A. indicus*; 16,306.0 ± 1705.1, *A. amyesii*; 14,596.8 ± 1120.4, *A. towneri*), indicating optimal amplification balance. This proportion was therefore adopted as the optimal primer ratio for the multiplex PCR.

### 3.3. Evaluation of Specific Detection Primers

**Figure 4:**
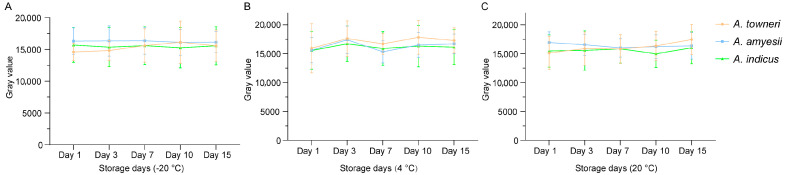
Primer stability under −20 °C, 4 °C, and 20 °C storages. Primer stability for detection of *A. towneri*, *A.amyesii*, and *A. indicus* under −20 °C (**A**), 4 °C (**B**), and 20 °C (**C**) storage conditions is presented, respectively.

Sensitivity was assessed by a two-fold serial dilution of a mixed genomic DNA template (initial concentration, 30 ng/µL) prepared from the three *tet*(X)-positive *Acinetobacter* species. As shown in Figure 3D, four specific amplicons of approximately 267 bp, 424 bp, 690 bp, and 990 bp were clearly visible at template concentrations ≥0.3 ng/µL. Below this threshold, the 424 bp, 690 bp, and 990 bp fragments were consistently undetectable. Consequently, the limit of detection for the multiplex PCR was established at 0.3 ng/µL genomic DNA, demonstrating satisfactory analytical sensitivity. To evaluate primer stability (Figure 4), the intensity of target amplicons was quantified during 15 days of storage at −20 °C (ranging from 14,596.8 ± 1120.4 to 16,366.4 ± 1728.1), 4 °C (ranging from 15,311.5 ± 1559.7 to 17,814.3 ± 2376.9), and 20 °C (ranging from 14,963.4 ± 1924.1 to 17,445.3 ± 2130.0). No significant decline in band intensity was observed under any condition, indicating that the multiplex-specific primers retain full activity across the tested temperature range and are sufficiently robust for routine deployment under varied storage scenarios.

Both genomic DNA and bacterial suspensions were applied for the next analyses. Briefly, screening of 151 non-duplicate bacterial genomes by the established multiplex PCR confirmed 45 *tet*(X)-positive *A. indicus*, 17 *tet*(X)-positive *A. amyesii*, and 8 *tet*(X)-positive *A. towneri* isolates, with the detection accuracy of 97.8%, 100%, and 100%, respectively (Table 2). All the others were negative except one *tet*(X)-positive *A. variabilis* (3.6%) misdiagnosed as *A. amyesii*. In addition, results of bacterial suspensions were highly consistent with those of genomic DNA, giving the accuracy of 93.5%, 100%, and 100% within two hours (Table 2). These data validate the practicability for the rapid and accurate identification of *tet*(X)-positive *A. indicus*, *A. amyesii*, and *A. towneri* by multiplex specific PCR.

## 4. Discussion

A major concern for global public health is the alarming propensity of *Acinetobacter* sp. pathogens to acquire genetic determinants for multidrug resistance and limited treatment options [4,6,30]. The bioinformatic findings of this study demonstrated that *Acinetobacter* species served as a reservoir for the mobile tigecycline resistance gene *tet*(X), with 20 variants (predominantly *tet*(X3)) having been detected therein. *A. indicus*, *A. amyesii*, and *A. towneri* were the predominant species among 390 *tet*(X)-mediated tigecycline-resistant *Acinetobacter* sp. isolates. In the era of One Health, *A. indicus* have been frequently identified in human, pig, chicken, goose, duck, pigeon, cattle, cow, migratory bird, water, soil, and waste materials since its first report in 2012 [14,31,32,33]. *A. towneri* has been detected in human, pig, chicken, cattle, water, soil, and activated sludge samples since its first report in 2003 [14,34,35,36,37,38]. *A. amyesii* has been sporadically found in pig, cow, water, soil, and dust samples since its first report in 2022 [14,39,40]. Despite the lack of detailed clinical infection data, *A. indicus*, *A. amyesii*, and *A. towneri* strains represent potential pathogenic microorganisms.

Furthermore, multiple antibiotic resistance genes conferring resistance to eight classes of antibiotics were collected in *tet*(X)-positive *Acinetobacter* spp. in this study, especially *A. indicus*, *A. amyesii*, and *A. towneri*. As previously reported, they were commonly resistant to tetracycline, tigecycline, eravacycline, omadacycline, florfenicol, trimethoprim/sulfamethoxazole, and ciprofloxacin [14,41]. Concurrently, the carbapenem resistance genes *bla*_NDM-1_ and *bla*_NDM-3_ were also identified in *tet*(X)-positive *A. indicus* and *A. towneri* strains from cow, pig, duck, goose, chicken, soil, and hospital wastewater samples in China and the Philippines [13,14,15,16,17,42]. Therefore, the development of rapid detection methods for monitoring *tet*(X)-positive MDR *A. indicus*, *A. amyesii*, and *A. towneri* is justified.

Given the urgent threat posed by the growing number of *tet*(X) variants, a series of multifaceted approaches has been undertaken to tackle this challenge. Specific primers against *tet*(X1), *tet*(X2), *tet*(X3), *tet*(X4), and *tet*(X5) were designed, enabling the development of multiplex PCR, SYBR green-based qPCR, and TaqMan-based qPCR assays [43,44,45]. Notably, a highly sensitive loop-mediated isothermal amplification assay with a visual orange to green dye was implemented for simultaneous detection of these variants [46]. A phenotypic detection method was established by coupling an acid-base indicator, bromocresol purple, with the degradation of eravacycline by *tet*(X3)- and *tet*(X4)-positive bacterial strains [47]. A one-tube recombinase polymerase amplification (RPA)-CRISPR-Cas12b system was developed for *tet*(X4)-positive strains [48]. Moreover, MALDI-TOF MS-based tests were utilized for rapid identification of Tet(X)-producing strains [49,50]. A liquid chromatography-tandem mass spectrometry (LC-MS/MS) method of metabolite ratios was developed to detect *tet*(X) in *Enterobacteriaceae* bacteria [51]. Continuous evolution of *tet*(X) variants highlights the need for ongoing methodological updates to ensure the detection coverage and accuracy, especially in diverse *Acinetobacter* species.

Traditional methods for *Acinetobacter* identification typically rely on the 16S rRNA sequencing, *rpoB* sequencing, *gyrB*-based multiplex PCR, ANI, phenotypic system VITEK 2, and MALDI-TOF MS, which often entail high economic costs and database dependencies [18,52,53]. In contrast, the multiplex PCR method established in this study demonstrated high specificity, sensitivity, and stability, while also being cost-effective, thereby facilitating rapid monitoring of the top three *tet*(X)-positive species (namely *A. indicus*, *A. amyesii*, and *A. towneri*). It cannot be ignored that *tet*(X)-positive *A. variabilis* may interfere with the detection accuracy of *tet*(X)-positive *A. amyesii*. Inevitably, several limitations may also exist in the clinical and environmental application. For example, false negatives or non-target results can occur with degraded nucleic acids or multiple bacterial templates in the complex background [54]. PCR sensitivity is limited in low-abundance pathogens within large-volume samples [55]. The detection technology cannot clearly differentiate live from dead microbes [56]. On the other hand, the diversity of *tet*(X)-positive *Acinetobacter* species is considerable, including clinically important *A. baumannii*, *A. pittii*, and *A. junii* [5,7,16,57]. To the best of our knowledge, the specific detection method targeting *tet*(X)-positive *A. indicus*, *A. amyesii*, and *A. towneri* was first reported, and the techniques for the remaining *Acinetobacter* species warrant further exploration.

## 5. Conclusions

In summary, this study emphasized the global prevalence status of tigecycline resistance *tet*(X) variants in complex *Acinetobacter* genomes. Combined pan-genome analyses with experimental validation, a multiplex PCR method was successfully established and optimized for the specific detection of three predominant *tet*(X)-positive *Acinetobacter* species, namely *A. indicus*, *A. amyesii*, and *A. towneri*. The experimental protocol demonstrated high sensitivity, high accuracy, and robust primer stability across different storage temperatures. According to clarifying the distribution landscape of *tet*(X) variants and enabling rapid identification, this method will provide a reliable and cost-effective tool for the future surveillance of *tet*(X)-mediated tigecycline-resistant *Acinetobacter* sp. pathogens.

## Figures and Tables

**Table 1:** PCR primers of predominant *tet*(X)-positive *Acinetobacter* species.

Primer	Sequence (5′-3′)	Size (bp)	Target
tetX-F	GCGGGATTGTTACAAACTTA	267	*tet*(X)
tetX-R	ATCTGCTGTTTCACTCG		
indicus-F	ATGCAATTAACCGATTATCCAG	424	*A. indicus*
indicus-R	CCAGATAATGCCCCACACT		
amyesii-F	GCCTATGTTTTTGACCCAAT	690	*A. amyesii*
amyesii-R	GCACCATAAAACCAATACC		
towneri-F	TGGGTAGATGTGTCACAGG	990	*A. towneri*
towneri-R	GGTATTCAAACCAATGACTGC		

**Table 2:** Multiplex PCR detection results.

Species	Number	Percentage (Positive Strains/Samples)	
		Bacterial Suspensions	Genomic DNA
*tet*(X)-positive strains	145	47.6% (69/145)	49% (71/145)
*A. indicus*	46	93.5% (43/46)	97.8% (45/46)
*A. amyesii*	17	100% (17/17)	100% (17/17)
*A. towneri*	8	100% (8/8)	100% (8/8)
*A. variabilis*	28	3.6% (1/28)	3.6% (1/28)
*A. schindleri*	5	0% (0/5)	0% (0/5)
*A. pseudolwoffii*	5	0% (0/5)	0% (0/5)
*A. sichuanensis*	2	0% (0/2)	0% (0/2)
*A. lwoffii*	1	0% (0/1)	0% (0/1)
*A. defluvii*	1	0% (0/1)	0% (0/1)
*E. coli*	9	0% (0/9)	0% (0/9)
*A. caviae*	1	0% (0/1)	0% (0/1)
*E. stercoris*	13	0% (0/13)	0% (0/13)
*M. tengzhouensis*	1	0% (0/1)	0% (0/1)
*M. odoratimimus*	5	0% (0/5)	0% (0/5)
*M. zaozhuangensis*	1	0% (0/1)	0% (0/1)
*M. faecalis*	2	0% (0/2)	0% (0/2)
*tet*(X)-negative strains	6	0% (0/6)	0% (0/6)
*E. coli*	2	0% (0/2)	0% (0/2)
*A. baumannii*	1	0% (0/1)	0% (0/1)
*A. baylyi*	1	0% (0/1)	0% (0/1)
*S. enterica*	1	0% (0/1)	0% (0/1)
*K. pneumoniae*	1	0% (0/1)	0% (0/1)

## Data Availability

The original contributions presented in this study are included in the article/Supplementary Materials. Further inquiries can be directed to the corresponding author.

## Supplementary Materials

The following supporting information can be downloaded at: https://www.mdpi.com/article/10.3390/microorganisms13112584/s1. Figure S1: Distribution of multiple antibiotic resistance genes in *tet*(X)-positive *Acinetobacter* sp. bacteria; Table S1: PCR reaction system in this study; Table S2: PCR reaction procedure in this study; Table S3: Bacterial information of *tet*(X)-positive *Acinetobacter* sp. strains.

## Abbreviations

The following abbreviations are used in this manuscript:

CRAb	Carbapenem-resistant *Acinetobacter baumannii*
MDR	Multidrug-resistant
qPCR	Quantitative real-time PCR
ANI	Average Nucleotide Identity
MALDI-TOF MS	Matrix-assisted laser desorption/ionization time-of-flight mass spectrometry
NCBI	National Center for Biotechnology Information
STs	Sequence Types
CARD	Comprehensive Antibiotic Resistance Database
RPA	Recombinase polymerase amplification
LC-MS/MS	Liquid chromatography-tandem mass spectrometry
