# Supplementary material for: Establishment of Specific Multiplex PCR Detection Methods for the Predominant tet(X)-Positive Acinetobacter Species
Source: Microorganisms. 2025 Nov 12;13(11):2584. doi: 10.3390/microorganisms13112584 (PMC12654350; doi:10.3390/microorganisms13112584)
Supplement: Supplementary file 1 [file microorganisms-13-02584-s001.zip › microorganisms-3965787-supplementary.pdf]

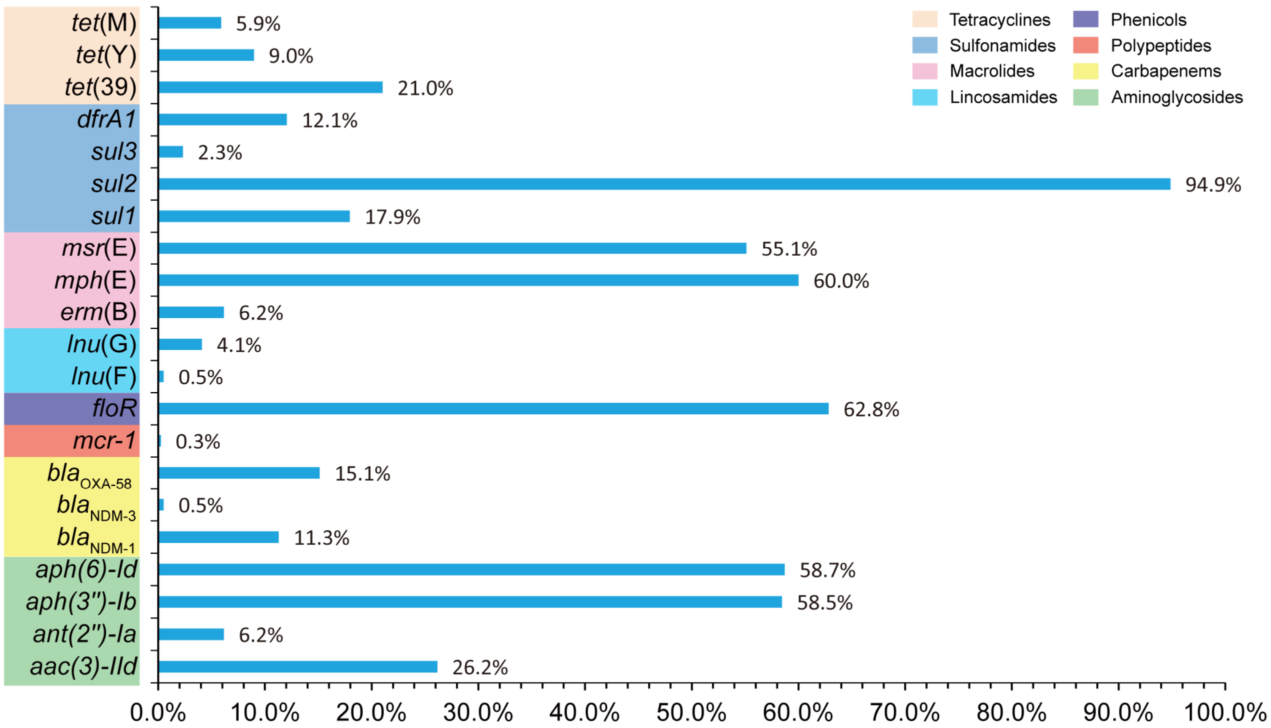

Figure S1. Distribution of multiple antibiotic resistance genes in *tet*(X)-positive *Acinetobacter* sp. bacteria. There are eight classes of antibiotic resistance genes marked in different colors.

Table S1. PCR reaction system in this study.

Composition	Volume (25 μL)
Sterile H_2O [a]	10.5 μL
Upstream primer	0.5 μL
Downstream primer	0.5 μL
2×Taq Master Mix	12.5 μL
PCR template	1 μL

[a] The volume of sterile water can be adjusted according to primers.

Table S2. PCR reaction procedure in this study.

Objective	Parameter
Bacterial genome/ suspension	(94 ℃, 5 min)+[(94 ℃, 30 s)+(53 ℃ [a], 30 s)+(72 ℃, 30 S)]×35+(72 ℃, 5 min)+ 4 ℃, ∞

[a] The annealing temperature can be adjusted to explore the optimal one.

Table S3. Bacterial information of *tet*(X)-positive *Acinetobacter* sp. strains.

Order	Strain	tet(X)	Species (ANI)	ST [a]	Year [a]	Country	Source	Accession number
1	XM9F202-2	tet(X3), tet(X15)	Acinetobacter variabilis	1999	2020	China	chicken	GCA_016607565.1
2	19110F47	tet(X4)	Acinetobacter towneri	1992	2019	China	pig	GCA_009676805.1
3	GXNN15X4	tet(X4)	Acinetobacter indicus	2017	2019	China	porcine	GCA_017309425.1
4	GXNN62X4	tet(X4)	Acinetobacter indicus	1945	2019	China	porcine	GCA_017309445.1
5	Q22-2	tet(X4)	Acinetobacter indicus	-	2017	China	migratory bird	GCA_013420625.1
6	Q278-1	tet(X4)	Acinetobacter indicus	-	2017	China	migratory bird	GCA_013420575.1
7	Q85-2	tet(X4)	Acinetobacter indicus	-	2017	China	migratory bird	GCA_013420555.1
8	Q186-3	tet(X4), tet(X6)	Acinetobacter indicus	-	2017	China	migratory bird	GCA_013420655.1
9	Q66-1	tet(X4), tet(X6)	Acinetobacter indicus	-	2017	China	migratory bird	GCA_013420565.1
10	BDT201	tet(X3), tet(X5.4), tet(X6)	Acinetobacter indicus	-	2020	China	porcine	GCA_022749635.1
11	149	tet(X3), tet(X6)	Taxon 47	-	2019	China	chicken	GCA_030329225.1
12	18QD2AZ28W	tet(X3), tet(X6)	Acinetobacter pseudolwoffii	-	2018	China	pig	GCA_009829005.1
13	18QD2AZ57W	tet(X3), tet(X6)	Acinetobacter johnsonii	-	2018	China	pig	GCA_009828985.1
14	205	tet(X6)	Acinetobacter towneri	1993	2019	China	pig	GCA_012220585.1
15	226-1	tet(X3), tet(X6)	Acinetobacter sichuanensis	-	2019	China	chicken	GCA_030329325.1
16	226-4	tet(X3), tet(X6)	Acinetobacter sichuanensis	-	2019	China	chicken	GCA_030329335.1
17	251-1	tet(X6)	Taxon 76	-	2019	China	chicken	GCA_030329305.1
18	256-1	tet(X6)	Taxon 76	-	2019	China	chicken	GCA_030329285.1
19	29FS20	tet(X5)	Acinetobacter baumannii	16	2017	China	duck	GCA_013394285.1
20	31FS3-2	tet(X6)	Acinetobacter baumannii	1093	2017	China	duck	GCA_013394265.1
21	AB1	tet(X6)	Acinetobacter baumannii	52	2009	China	homo sapiens	GCA_014218985.1
22	AB17H194	tet(X5)	Acinetobacter pittii	795	2017	China	homo sapiens	GCA_008694085.1
23	ABF9692	tet(X5.3)	Acinetobacter baumannii	23	2017	China	duck	GCA_012974585.1

24	AT185	tet(X6)	Acinetobacter towneri	-	2019	China	pig	GCA_020280985.1
25	AT208	tet(X6)	Acinetobacter towneri	1993	2019	China	pig	GCA_020280905.1
26	AT232	tet(X6)	Acinetobacter towneri	-	2019	China	pig	GCA_022700695.1
27	AT235	tet(X6)	Acinetobacter towneri	-	2019	China	pig	GCA_022700715.1
28	CMG3-2	tet(X3), tet(X6)	Acinetobacter indicus	-	2017	China	goose	GCA_010917815.1
29	DF25-2	tet(X3), tet(X6)	Acinetobacter indicus	-	2019	China	chicken	GCA_030329345.1
30	DF25-5-2	tet(X3), tet(X6)	Acinetobacter indicus	-	2019	China	chicken	GCA_030329385.1
31	DF29-5	tet(X3), tet(X6)	Acinetobacter towneri	-	2019	China	chicken	GCA_030329035.1
32	FS42-2	tet(X3), tet(X6)	Acinetobacter indicus	1946	2017	China	duck	GCA_010918035.1
33	GFQ9D191M	tet(X6)	Acinetobacter variabilis	-	2019	China	duck	GCA_011174875.1
34	GFQ9D192M	tet(X6)	Acinetobacter variabilis	-	2019	China	duck	GCA_011192225.1
35	HZE23-1	tet(X3), tet(X6)	Acinetobacter schindleri	1976	2018	China	goose	GCA_010918635.1
36	HZE30-1	tet(X3), tet(X6)	Acinetobacter schindleri	1797	2018	China	goose	GCA_010918895.1
37	HZE33-1	tet(X3), tet(X6)	Acinetobacter schindleri	1977	2018	China	goose	GCA_010919095.1
38	LHC22-2	tet(X6)	Acinetobacter baumannii	-	2020	China	chicken	GCA_020271605.1
39	LYS68A	tet(X3), tet(X6)	Acinetobacter indicus	2009	2019	China	guinea fowl	GCA_017132735.1
40	MMS9-2	tet(X3), tet(X6)	Acinetobacter indicus	2014	2018	China	soil	GCA_010918195.1
41	N118-2	tet(X6)	Acinetobacter towneri	-	2019	China	cattle	GCA_030325685.1
42	N169	tet(X3), tet(X6)	Acinetobacter indicus	-	2019	China	cattle	GCA_030325545.1
43	N195	tet(X3), tet(X6)	Acinetobacter indicus	-	2019	China	cattle	GCA_030325505.1
44	R933-2	tet(X3), tet(X6)	Acinetobacter sichuanensis	-	2019	China	homo sapiens	GCA_030325485.1
45	R960	tet(X3), tet(X6)	Acinetobacter johnsonii	-	2019	China	homo sapiens	GCA_030325455.1
46	S1180-10	tet(X27.3)	Acinetobacter indicus	-	2019	Czech Republic	Gallus gallus	GCA_024129835.1
47	S1180-5	tet(X27.3)	Acinetobacter indicus	-	2019	Czech Republic	Gallus gallus	GCA_024129655.1
48	S249-3	tet(X27.3)	Acinetobacter indicus	-	2019	Czech Republic	Gallus gallus	GCA_024129665.1

49	SCsl29	tet(X6)	Acinetobacter variabilis	2000	-	China	pig	GCA_009822135.1
50	SH20PTE14	tet(X3), tet(X6)	Acinetobacter amyesii	-	2020	China	pig	GCA_021391555.1
51	TB2-2B	tet(X3), tet(X6)	Acinetobacter variabilis	-	2022	China	soil	GCA_030518655.1
52	X4-107	tet(X6)	Acinetobacter baumannii	724	2020	China	homo sapiens	GCA_029774295.1
53	X4-136	tet(X6)	Acinetobacter baumannii	724	2020	China	homo sapiens	GCA_029774275.1
54	X4-201	tet(X6)	Acinetobacter baumannii	724	2020	China	homo sapiens	GCA_029774315.1
55	X4-300	tet(X6)	Acinetobacter baumannii	724	2020	China	homo sapiens	GCA_029774235.1
56	X4-584	tet(X6)	Acinetobacter baumannii	724	2020	China	homo sapiens	GCA_029774335.1
57	X4-65	tet(X6)	Acinetobacter baumannii	724	2020	China	homo sapiens	GCA_029814975.1
58	X4-705	tet(X6)	Acinetobacter baumannii	724	2020	China	homo sapiens	GCA_029774355.1
59	XMC5X702	tet(X3), tet(X6)	Acinetobacter pseudolwoffii	1969	2020	China	chicken	GCA_020271765.1
60	YC103	tet(X6.7)	Acinetobacter baumannii	1093	2019	China	duck	GCA_021729465.1
61	YH01004	tet(X3), tet(X6)	Acinetobacter towneri	-	2017	China	pig	GCA_013420455.1
62	YH01005	tet(X3), tet(X6)	Acinetobacter amyesii	-	2017	China	sewage	GCA_013420465.1
63	YH01006	tet(X3), tet(X6)	Acinetobacter pseudolwoffii	-	2017	China	pig	GCA_013420445.1
64	YH01010	tet(X3), tet(X6)	Acinetobacter amyesii	-	2017	China	pig	GCA_013420775.1
65	YH01013	tet(X3), tet(X6)	Acinetobacter towneri	-	2017	China	pig	GCA_013420795.1
66	YH01015	tet(X3), tet(X6)	Acinetobacter schindleri	-	2017	China	pig	GCA_013420325.1
67	YH12021	tet(X3), tet(X6)	Acinetobacter towneri	-	2017	China	sewage	GCA_013420145.1
68	YH12061	tet(X3), tet(X6)	Acinetobacter amyesii	-	2017	China	pig	GCA_013419745.1
69	YH12062	tet(X3), tet(X6)	Acinetobacter amyesii	-	2017	China	pig	GCA_013419725.1
70	YH12068	tet(X3), tet(X6)	Acinetobacter amyesii	-	2017	China	pig	GCA_013419635.1
71	YH12075	tet(X3), tet(X6)	Acinetobacter towneri	-	2015	China	pig	GCA_013419545.1
72	YH12089	tet(X3), tet(X6)	Acinetobacter indicus	-	2016	China	pig	GCA_013419335.1
73	YH12094	tet(X3), tet(X6)	Acinetobacter amyesii	-	2017	China	pig	GCA_013419295.1

74	YH12109	tet(X3), tet(X6)	Acinetobacter amyesii	-	2017	China	pig	GCA_013419025.1
75	YH12110	tet(X3), tet(X6)	Acinetobacter indicus	-	2017	China	pig	GCA_013419035.1
76	YH12132	tet(X3), tet(X6)	Acinetobacter indicus	-	2017	China	pig	GCA_013418645.1
77	YH12133	tet(X3), tet(X6)	Acinetobacter indicus	-	2017	China	pig	GCA_013418575.1
78	YH12138	tet(X3), tet(X6)	Acinetobacter amyesii	-	2017	China	pig	GCA_013418445.1
79	YH12139	tet(X3), tet(X6)	Acinetobacter indicus	-	2017	China	pig	GCA_013418455.1
80	YH12147	tet(X3), tet(X6)	Acinetobacter indicus	-	2017	China	pig	GCA_013418365.1
81	YH12200	tet(X3), tet(X6)	Acinetobacter pseudolwoffii	-	2017	China	soil	GCA_013418175.1
82	YH12206	tet(X3), tet(X5.3)	Taxon 76	1964	2017	China	pig	GCA_013418115.1
83	YH12207	tet(X3), tet(X5.3)	Taxon 76	1964	2017	China	pig	GCA_013418085.1
84	YH12209	tet(X3), tet(X5.3)	Taxon 76	1964	2017	China	soil	GCA_013418065.1
85	YH12210	tet(X3), tet(X5.3)	Taxon 76	1964	2017	China	soil	GCA_013418025.1
86	YH12219	tet(X3), tet(X6)	Acinetobacter amyesii	-	2017	China	pig	GCA_013417875.1
87	YH12235	tet(X3), tet(X6)	Acinetobacter variabilis	-	2016	China	pig	GCA_013417705.1
88	YH12250	tet(X3), tet(X6)	Acinetobacter amyesii	-	2017	China	pig	GCA_013417435.1
89	YH16032	tet(X3), tet(X6)	Taxon 58	-	2016	China	dust	GCA_013417345.1
90	YH16038	tet(X3), tet(X6)	Acinetobacter variabilis	-	2016	China	soil	GCA_013417275.1
91	YH16039	tet(X3), tet(X6)	Taxon 32	-	2016	China	sewage	GCA_013417255.1
92	YH16049	tet(X3), tet(X6)	Acinetobacter variabilis	-	2016	China	pig	GCA_013417175.1
93	YH16051	tet(X3), tet(X6)	Acinetobacter towneri	-	2016	China	pig	GCA_013417135.1
94	YH16052	tet(X3), tet(X6)	Acinetobacter lwoffii	-	2016	China	pig	GCA_013417155.1
95	YH1901134	tet(X3), tet(X6)	Acinetobacter amyesii	-	2015	China	pig	GCA_013416965.1
96	IHIT27599	tet(X6.6)	Acinetobacter indicus	-	2014	Germany	calf	GCA_001922645.1
97	IHIT31230	tet(X6.6)	Acinetobacter indicus	-	2016	Germany	cattle	GCA_002938815.1
98	IHIT33295	tet(X6.6)	Acinetobacter indicus	-	2015	Germany	cattle	GCA_002938905.1

99	S289-4	tet(X6.6)	Acinetobacter indicus	-	2019	Czech Republic	Gallus gallus	GCA_024129795.1
100	JF351-2	tet(X27.4)	Acinetobacter bereziniae	-	2019	China	chicken	GCA_030329445.1
101	10FS3-1	tet(X3)	Taxon 83	-	2015	China	pig	GCA_013420695.1
102	11J360	tet(X3)	Acinetobacter nosocomialis	68	2012	China	homo sapiens	GCA_030322945.1
103	2021CK-01300	tet(X3)	Acinetobacter baumannii	422	2021	USA	homo sapiens	GCA_025258765.1
104	2021CK-01332	tet(X3)	Acinetobacter baumannii	422	2021	USA	homo sapiens	GCA_025258785.1
105	2021CK-01333	tet(X3)	Acinetobacter baumannii	422	2021	USA	homo sapiens	GCA_025258805.1
106	2021CK-01335	tet(X3)	Acinetobacter baumannii	422	2021	USA	homo sapiens	GCA_025258845.1
107	2021CK-01407	tet(X3)	Acinetobacter baumannii	422	2021	USA	homo sapiens	GCA_025369955.1
108	2021CK-01408	tet(X3)	Acinetobacter baumannii	422	2021	USA	homo sapiens	GCA_025258705.1
109	2021CK-01409	tet(X3)	Acinetobacter baumannii	422	2021	USA	homo sapiens	GCA_025258745.1
110	28F	tet(X3)	Acinetobacter nosocomialis	71	-	Colombia	homo sapiens	GCA_000529215.1
111	42F	tet(X3)	Acinetobacter pittii	63	-	Colombia	homo sapiens	GCA_000529235.1
112	4300STDY7045708	tet(X3)	Acinetobacter baumannii	578	2016	Thailand	homo sapiens	GCA_900494955.1
113	4300STDY7045823	tet(X3)	Acinetobacter baumannii	578	2016	Thailand	homo sapiens	GCA_900496045.1
114	80-1-2	tet(X3)	Acinetobacter indicus	-	2019	China	cow	GCA_012278735.1
115	94-2	tet(X3)	Acinetobacter indicus	2012	2018	China	cow	GCA_012278715.1
116	A1	tet(X3)	Taxon 91	-	2019	China	cow	GCA_012371315.1
117	A2	tet(X3)	Taxon 91	-	2019	China	cow	GCA_012371325.1
118	A3	tet(X3)	Taxon 91	-	2019	China	cow	GCA_012371415.1
119	AC1	tet(X3)	Acinetobacter amyesii	-	2018	China	cow	GCA_012371365.1
120	AI1	tet(X3)	Acinetobacter indicus	2012	2019	China	cow	GCA_012371455.1
121	AI10	tet(X3)	Acinetobacter indicus	-	2018	China	cow	GCA_012371195.1
122	AI11	tet(X3)	Acinetobacter indicus	2012	2018	China	cow	GCA_012366895.1
123	AI12	tet(X3)	Acinetobacter indicus	-	2018	China	cow	GCA_012371165.1

124	AI13	tet(X3)	Acinetobacter indicus	-	2018	China	cow	GCA_012366855.1
125	AI14	tet(X3)	Acinetobacter indicus	-	2018	China	cow	GCA_012366815.1
126	AI15	tet(X3)	Acinetobacter indicus	2012	2018	China	cow	GCA_012366785.1
127	AI18	tet(X3)	Acinetobacter indicus	-	2018	China	cow	GCA_012366735.1
128	AI19	tet(X3)	Acinetobacter indicus	-	2019	China	cow	GCA_012366715.1
129	AI2	tet(X3)	Acinetobacter indicus	-	2018	China	cow	GCA_012366935.1
130	AI20	tet(X3)	Acinetobacter indicus	-	2019	China	cow	GCA_012366755.1
131	AI21	tet(X3)	Acinetobacter indicus	-	2019	China	cow	GCA_012366655.1
132	AI22	tet(X3)	Acinetobacter indicus	-	2018	China	cow	GCA_012366665.1
133	AI23	tet(X3)	Acinetobacter indicus	-	2018	China	cow	GCA_012366675.1
134	AI24	tet(X3)	Acinetobacter indicus	-	2018	China	cow	GCA_012366635.1
135	AI25	tet(X3)	Acinetobacter indicus	2012	2018	China	cow	GCA_012371125.1
136	AI26	tet(X3)	Acinetobacter indicus	-	2018	China	cow	GCA_012371095.1
137	AI27	tet(X3)	Acinetobacter indicus	-	2018	China	cow	GCA_012371115.1
138	AI28	tet(X3)	Acinetobacter indicus	2012	2018	China	cow	GCA_012371045.1
139	AI29	tet(X3)	Acinetobacter indicus	-	2019	China	cow	GCA_012371035.1
140	AI3	tet(X3)	Acinetobacter indicus	-	2018	China	cow	GCA_012371255.1
141	AI30	tet(X3)	Acinetobacter indicus	2012	2019	China	cow	GCA_012371275.1
142	AI31	tet(X3)	Acinetobacter indicus	-	2019	China	cow	GCA_012370955.1
143	AI32	tet(X3)	Acinetobacter indicus	-	2018	China	cow	GCA_012371015.1
144	AI33	tet(X3)	Acinetobacter indicus	-	2018	China	cow	GCA_012371075.1
145	AI34	tet(X3)	Acinetobacter indicus	-	2018	China	cow	GCA_012371295.1
146	AI35	tet(X3)	Acinetobacter indicus	2012	2018	China	cow	GCA_012370815.1
147	AI37	tet(X3)	Acinetobacter indicus	-	2018	China	cow	GCA_012370935.1
148	AI38	tet(X3)	Acinetobacter indicus	-	2019	China	cow	GCA_012370915.1

149	AI39	tet(X3)	Acinetobacter indicus	-	2018	China	cow	GCA_012370975.1
150	AI4	tet(X3)	Acinetobacter indicus	-	2018	China	cow	GCA_012366955.1
151	AI40	tet(X3)	Acinetobacter indicus	2012	2019	China	cow	GCA_012370895.1
152	AI41	tet(X3)	Acinetobacter indicus	2012	2018	China	cow	GCA_012370875.1
153	AI5	tet(X3)	Acinetobacter indicus	-	2018	China	cow	GCA_012371235.1
154	AI6	tet(X3)	Acinetobacter indicus	-	2019	China	cow	GCA_012366875.1
155	AI7	tet(X3)	Acinetobacter indicus	-	2018	China	cow	GCA_012371205.1
156	AI8	tet(X3)	Acinetobacter indicus	-	2018	China	cow	GCA_012366885.1
157	AI9	tet(X3)	Acinetobacter indicus	-	2018	China	cow	GCA_012371155.1
158	AJ_351	tet(X3)	Acinetobacter junii	1950	2016	Pakistan	washroom sink	GCA_003939335.2
159	AJ1	tet(X3)	Taxon 71	-	2018	China	cow	GCA_012371395.1
160	AJ-1	tet(X3)	Taxon 71	-	2018	China	cow	GCA_013186005.1
161	Ajun-H1-2	tet(X3)	Acinetobacter junii	-	2004	Israel	homo sapiens	GCA_035798055.1
162	AN1	tet(X3)	Acinetobacter nosocomialis	279	2010	China	homo sapiens	GCA_014218925.1
163	AT1	tet(X3)	Taxon 91	-	2018	China	cow	GCA_012371355.1
164	AT181	tet(X3)	Acinetobacter towneri	1994	2019	China	pig	GCA_020281045.1
165	AT184	tet(X3)	Acinetobacter towneri	1994	2019	China	pig	GCA_020281145.1
166	AT200	tet(X3)	Acinetobacter towneri	-	2019	China	pig	GCA_020281105.1
167	AT209	tet(X3)	Acinetobacter towneri	1994	2019	China	pig	GCA_020281015.1
168	AT211	tet(X3)	Acinetobacter towneri	1994	2019	China	pig	GCA_020281005.1
169	AT213	tet(X3)	Acinetobacter towneri	1994	2019	China	pig	GCA_020280965.1
170	AT214	tet(X3)	Acinetobacter towneri	1994	2019	China	pig	GCA_020280915.1
171	AT216	tet(X3)	Acinetobacter towneri	1994	2019	China	pig	GCA_020281085.1
172	AT217	tet(X3)	Acinetobacter towneri	1994	2019	China	pig	GCA_020281065.1
173	B18	tet(X3)	Acinetobacter indicus	-	2016	China	pigeon	GCA_010918375.1

174	BDT2044	tet(X3), tet(X3.3), tet(X3.4), tet(X3.5), tet(X3.6)	Acinetobacter variabilis	-	2020	China	pig	GCA_022749615.1
175	BDT2076	tet(X3)	Acinetobacter indicus	-	2020	China	chicken	GCA_022749595.1
176	BM4623	tet(X2)	Acinetobacter pittii	-	2017	China	homo sapiens	GCA_029873195.1
177	C15	tet(X3)	Acinetobacter indicus	1946	2015	China	pig	GCA_013420645.1
178	DF16-4	tet(X3)	Acinetobacter towneri	-	2019	China	chicken	GCA_030329245.1
179	DF23-4	tet(X3)	Acinetobacter towneri	-	2019	China	chicken	GCA_030329235.1
180	DF31-4-1	tet(X3)	Acinetobacter towneri	-	2019	China	chicken	GCA_030329185.1
181	DF34-4	tet(X3)	Acinetobacter towneri	-	2019	China	chicken	GCA_030329165.1
182	DF3-5	tet(X3)	Acinetobacter towneri	-	2019	China	chicken	GCA_030329205.1
183	DF35-4	tet(X3)	Acinetobacter towneri	-	2019	China	chicken	GCA_030329125.1
184	DF38-4	tet(X3)	Acinetobacter towneri	-	2019	China	chicken	GCA_030329145.1
185	DF39-2	tet(X3)	Acinetobacter towneri	-	2019	China	chicken	GCA_030329105.1
186	DF40-4	tet(X3)	Acinetobacter towneri	-	2019	China	chicken	GCA_030329085.1
187	DF49-4	tet(X3)	Acinetobacter towneri	-	2019	China	chicken	GCA_030329065.1
188	ESKAPEE pathogen	tet(X3)	Acinetobacter baumannii	578	2017	Thailand	homo sapiens	GCA_023783775.1
189	GD03717	tet(X3)	Acinetobacter johnsonii	-	2018	Pakistan	sink	GCA_029843655.1
190	GD03725	tet(X3)	Acinetobacter johnsonii	-	2018	Pakistan	sink	GCA_029843525.1
191	GD03738	tet(X3)	Acinetobacter johnsonii	-	2018	Pakistan	sink	GCA_029843265.1
192	GD03910	tet(X3)	Acinetobacter junii	1950	2018	Pakistan	sink	GCA_029839935.1
193	GD03920	tet(X3)	Acinetobacter johnsonii	-	2018	Pakistan	sink	GCA_029839745.1
194	GX3	tet(X3)	Acinetobacter towneri	1990	2019	China	porcine	GCA_017498585.1
195	GX5	tet(X3)	Acinetobacter towneri	1990	2019	China	porcine	GCA_017498605.1
196	GX7	tet(X3)	Acinetobacter towneri	1991	2019	China	porcine	GCA_017498625.1

197	HY20	tet(X3)	Acinetobacter indicus	-	2018	China	duck	GCA_009296145.1
198	JNE3-2	tet(X3)	Acinetobacter faecalis	-	2021	China	milking environment	GCA_023155375.1
199	JNE5	tet(X3)	Acinetobacter faecalis	-	2021	China	milking environment	GCA_023158935.1
200	JXA13	tet(X3)	Acinetobacter pittii	795	2019	China	dog	GCA_016804045.1
201	MIN-010	tet(X3)	Acinetobacter baumannii	132	2021	Ghana	homo sapiens	GCA_021460405.1
202	MRSN22112	tet(X3)	Acinetobacter baumannii	193	2013	Peru	homo sapiens	GCA_006493975.1
203	N105-3	tet(X3)	Acinetobacter indicus	-	2019	China	cattle	GCA_030325675.1
204	N11-2	tet(X3)	Acinetobacter pseudolwoffii	-	2019	China	cattle	GCA_030325955.1
205	N123	tet(X3)	Acinetobacter indicus	-	2019	China	cattle	GCA_030325645.1
206	N160	tet(X3)	Acinetobacter indicus	-	2019	China	cattle	GCA_030325585.1
207	N167-2	tet(X3)	Acinetobacter indicus	-	2019	China	cattle	GCA_030325605.1
208	N17-1	tet(X3)	Acinetobacter pseudolwoffii	-	2019	China	cattle	GCA_030326035.1
209	N178-1	tet(X3)	Acinetobacter indicus	-	2019	China	cattle	GCA_030325565.1
210	N185	tet(X3)	Acinetobacter indicus	-	2019	China	cattle	GCA_030325665.1
211	N186-2	tet(X3)	Acinetobacter indicus	-	2019	China	cattle	GCA_030325525.1
212	N26-2	tet(X3)	Acinetobacter indicus	-	2019	China	cattle	GCA_030327775.1
213	N31-1	tet(X3)	Taxon 91	-	2019	China	cattle	GCA_030327765.1
214	N41-1	tet(X3.12)	Acinetobacter indicus	2012	2019	China	cattle	GCA_030325925.1
215	N43-2	tet(X3)	Acinetobacter indicus	-	2019	China	cattle	GCA_030325865.1
216	N49-2	tet(X3.12)	Acinetobacter indicus	2012	2019	China	cattle	GCA_030325905.1
217	N53-1	tet(X3)	Acinetobacter indicus	-	2019	China	cattle	GCA_030325845.1
218	N69-2	tet(X3)	Acinetobacter indicus	-	2019	China	cattle	GCA_030325765.1
219	N7-2	tet(X3)	Acinetobacter pseudolwoffii	-	2019	China	cattle	GCA_030325985.1
220	N86-2	tet(X3)	Acinetobacter indicus	-	2019	China	cattle	GCA_030325745.1
221	N93-1	tet(X3.12)	Acinetobacter indicus	2012	2019	China	cattle	GCA_030325725.1

222	N9-4	tet(X3)	Acinetobacter indicus	-	2019	China	cattle	GCA_030325945.1
223	NCGM 202	tet(X3), tet(X3.11)	Acinetobacter baumannii	578	2011	Viet Nam	homo sapiens	GCA_016502775.1
224	PW12	tet(X3)	Acinetobacter baumannii	132	2021	Ghana	hospital surface	GCA_020982665.1
225	R820	tet(X3)	Acinetobacter pittii	-	2018	China	homo sapiens	GCA_021385975.1
226	RF15A	tet(X3)	Taxon 83	-	2017	Ireland	hospital pen floor	GCA_007570885.1
227	RF15B	tet(X3)	Taxon 83	-	2017	Ireland	hospital pen floor	GCA_007558235.1
228	T167	tet(X3)	Acinetobacter pittii	655	2010	Thailand	homo sapiens	GCA_000805655.1
229	T820	tet(X3)	Acinetobacter pittii	-	2018	China	homo sapiens	GCA_021388455.1
230	T822-1	tet(X3)	Acinetobacter pittii	-	2018	China	homo sapiens	GCA_021388445.1
231	TQ04	tet(X3)	Acinetobacter indicus	2013	2017	China	cow	GCA_009914415.1
232	TQ18	tet(X3.10)	Acinetobacter indicus	2016	2017	China	cow	GCA_009914435.1
233	TQ23	tet(X3)	Acinetobacter indicus	2013	2017	China	cow	GCA_009914475.1
234	WB3	tet(X3)	Acinetobacter terrestris	-	2021	Canada	feedlot water bowl	GCA_030127305.1
235	WB5	tet(X3)	Acinetobacter pseudolwoffii	-	2021	Canada	feedlot water bowl	GCA_029887885.2
236	XG01	tet(X3)	Acinetobacter indicus	2015	2017	China	cow	GCA_009914375.1
237	XG03	tet(X3)	Acinetobacter indicus	-	2017	China	cow	GCA_009914395.1
238	YH01002	tet(X3)	Acinetobacter towneri	-	2017	China	pig	GCA_013420545.1
239	YH01003	tet(X3)	Acinetobacter towneri	-	2017	China	pig	GCA_013420525.1
240	YH01007	tet(X3)	Acinetobacter towneri	-	2017	China	pig	GCA_013420425.1
241	YH01008	tet(X3)	Acinetobacter pseudolwoffii	-	2017	China	pig	GCA_013420435.1
242	YH01009	tet(X3)	Acinetobacter amyesii	-	2017	China	pig	GCA_013420765.1
243	YH01011	tet(X3)	Acinetobacter towneri	-	2017	China	pig	GCA_013420785.1
244	YH01012	tet(X3)	Acinetobacter towneri	-	2017	China	pig	GCA_013420805.1
245	YH01016	tet(X3)	Taxon 32	-	2017	China	pig	GCA_013420305.1
246	YH01018	tet(X3)	Taxon 32	-	2017	China	pig	GCA_013420295.1

247	YH01020	tet(X3)	Acinetobacter amyesii	-	2017	China	pig	GCA_013420275.1
248	YH01021	tet(X3)	Acinetobacter lwoffii	-	2017	China	dust	GCA_013420255.1
249	YH01022	tet(X3)	Taxon 32	-	2017	China	soil	GCA_013420235.1
250	YH01024	tet(X3)	Acinetobacter amyesii	-	2017	China	pig	GCA_013420195.1
251	YH01025	tet(X3)	Acinetobacter indicus	-	2017	China	pig	GCA_013420185.1
252	YH01026	tet(X3)	Acinetobacter towneri	-	2017	China	pig	GCA_013420175.1
253	YH12023	tet(X3)	Acinetobacter amyesii	-	2017	China	pig	GCA_013420135.1
254	YH12025	tet(X3)	Acinetobacter amyesii	-	2017	China	dust	GCA_013420075.1
255	YH12027	tet(X3)	Acinetobacter amyesii	-	2017	China	pig	GCA_013420085.1
256	YH12029	tet(X3)	Acinetobacter amyesii	-	2017	China	pig	GCA_013420095.1
257	YH12035	tet(X3)	Acinetobacter variabilis	-	2017	China	dust	GCA_013419985.1
258	YH12036	tet(X3)	Acinetobacter amyesii	-	2017	China	pig	GCA_013419995.1
259	YH12039	tet(X3)	Acinetobacter amyesii	-	2017	China	pig	GCA_013420015.1
260	YH12040	tet(X3)	Acinetobacter amyesii	-	2017	China	pig	GCA_013420005.1
261	YH12041	tet(X3)	Acinetobacter variabilis	-	2017	China	pig	GCA_013419975.1
262	YH12043	tet(X3)	Acinetobacter variabilis	-	2017	China	pig	GCA_013419915.1
263	YH12045	tet(X3)	Acinetobacter amyesii	-	2017	China	pig	GCA_013419925.1
264	YH12046	tet(X3)	Acinetobacter variabilis	-	2017	China	pig	GCA_013419905.1
265	YH12049	tet(X3)	Acinetobacter amyesii	-	2017	China	pig	GCA_013419875.1
266	YH12052	tet(X3)	Acinetobacter amyesii	-	2017	China	pig	GCA_013419835.1
267	YH12054	tet(X3)	Acinetobacter amyesii	-	2017	China	pig	GCA_013419795.1
268	YH12057	tet(X3)	Acinetobacter amyesii	-	2017	China	pig	GCA_013419845.1
269	YH12058	tet(X3)	Acinetobacter towneri	-	2017	China	pig	GCA_013419805.1
270	YH12063	tet(X3)	Acinetobacter amyesii	-	2017	China	pig	GCA_013419715.1
271	YH12064	tet(X3)	Acinetobacter amyesii	-	2017	China	soil	GCA_013419735.1

272	YH12066	tet(X3)	Acinetobacter amyesii	-	2017	China	pig	GCA_013419685.1
273	YH12069	tet(X3)	Acinetobacter amyesii	-	2017	China	pig	GCA_013419675.1
274	YH12070	tet(X3)	Acinetobacter amyesii	-	2017	China	pig	GCA_013419625.1
275	YH12071	tet(X3)	Acinetobacter indicus	-	2017	China	pig	GCA_013419615.1
276	YH12072	tet(X3)	Acinetobacter variabilis	-	2017	China	pig	GCA_013419565.1
277	YH12073	tet(X3)	Acinetobacter amyesii	-	2017	China	pig	GCA_013419535.1
278	YH12076	tet(X3)	Acinetobacter amyesii	-	2015	China	pig	GCA_013419525.1
279	YH12079	tet(X3)	Acinetobacter amyesii	-	2015	China	pig	GCA_013419515.1
280	YH12080	tet(X3)	Acinetobacter amyesii	-	2015	China	pig	GCA_013419455.1
281	YH12081	tet(X3)	Acinetobacter amyesii	-	2015	China	pig	GCA_013419465.1
282	YH12082	tet(X3)	Acinetobacter amyesii	-	2015	China	pig	GCA_013419425.1
283	YH12083	tet(X3)	Acinetobacter amyesii	-	2015	China	pig	GCA_013419445.1
284	YH12085	tet(X3)	Acinetobacter amyesii	-	2015	China	pig	GCA_013419415.1
285	YH12086	tet(X3)	Acinetobacter amyesii	-	2015	China	pig	GCA_013419345.1
286	YH12087	tet(X3)	Acinetobacter amyesii	-	2015	China	pig	GCA_013419355.1
287	YH12090	tet(X3)	Acinetobacter indicus	-	2016	China	pig	GCA_013419325.1
288	YH12091	tet(X3)	Acinetobacter indicus	-	2016	China	pig	GCA_013419315.1
289	YH12095	tet(X3)	Acinetobacter towneri	-	2017	China	pig	GCA_013419245.1
290	YH12096	tet(X3)	Acinetobacter amyesii	-	2017	China	pig	GCA_013419215.1
291	YH12097	tet(X3)	Acinetobacter amyesii	-	2017	China	pig	GCA_013419225.1
292	YH12098	tet(X3)	Acinetobacter amyesii	-	2017	China	pig	GCA_013419235.1
293	YH12099	tet(X3)	Acinetobacter towneri	-	2017	China	pig	GCA_013419195.1
294	YH12100	tet(X3)	Acinetobacter pseudolwoffii	-	2017	China	pig	GCA_013419135.1
295	YH12101	tet(X3)	Acinetobacter indicus	-	2017	China	pig	GCA_013419145.1
296	YH12102	tet(X3)	Acinetobacter pseudolwoffii	-	2017	China	pig	GCA_013419125.1

297	YH12103	tet(X3)	Acinetobacter pseudolwoffii	-	2017	China	pig	GCA_013419115.1
298	YH12105	tet(X3)	Acinetobacter pseudolwoffii	-	2017	China	pig	GCA_013419095.1
299	YH12106	tet(X3)	Acinetobacter pseudolwoffii	-	2017	China	pig	GCA_013418995.1
300	YH12108	tet(X3)	Acinetobacter variabilis	-	2017	China	pig	GCA_013419055.1
301	YH12111	tet(X3)	Acinetobacter indicus	-	2017	China	pig	GCA_013419015.1
302	YH12112	tet(X3)	Acinetobacter indicus	-	2017	China	pig	GCA_013418945.1
303	YH12113	tet(X3)	Acinetobacter indicus	-	2017	China	pig	GCA_013418915.1
304	YH12114	tet(X3)	Acinetobacter indicus	-	2017	China	pig	GCA_013418935.1
305	YH12115	tet(X3)	Acinetobacter indicus	-	2017	China	pig	GCA_013418925.1
306	YH12116	tet(X3)	Acinetobacter indicus	-	2017	China	pig	GCA_013418895.1
307	YH12117	tet(X3)	Acinetobacter indicus	-	2017	China	pig	GCA_013418865.1
308	YH12118	tet(X3)	Acinetobacter indicus	-	2017	China	pig	GCA_013418835.1
309	YH12119	tet(X3)	Acinetobacter indicus	-	2017	China	pig	GCA_013418855.1
310	YH12120	tet(X3)	Acinetobacter towneri	-	2017	China	pig	GCA_013418795.1
311	YH12123	tet(X3)	Acinetobacter amyesii	-	2017	China	pig	GCA_013418805.1
312	YH12124	tet(X3)	Acinetobacter towneri	-	2017	China	pig	GCA_013418735.1
313	YH12125	tet(X3)	Acinetobacter towneri	-	2017	China	pig	GCA_013418755.1
314	YH12126	tet(X3)	Acinetobacter amyesii	-	2017	China	pig	GCA_013418745.1
315	YH12127	tet(X3)	Acinetobacter towneri	-	2017	China	pig	GCA_013418695.1
316	YH12128	tet(X3)	Acinetobacter amyesii	-	2017	China	pig	GCA_013418655.1
317	YH12129	tet(X3)	Acinetobacter amyesii	-	2017	China	pig	GCA_013418685.1
318	YH12131	tet(X3)	Acinetobacter towneri	-	2017	China	pig	GCA_013418635.1
319	YH12134	tet(X3)	Acinetobacter indicus	-	2017	China	soil	GCA_013418555.1
320	YH12135	tet(X3)	Acinetobacter towneri	-	2017	China	sewage	GCA_013418565.1
321	YH12136	tet(X3)	Acinetobacter towneri	-	2017	China	sewage	GCA_013418545.1

322	YH12137	tet(X3)	Acinetobacter variabilis	-	2017	China	pig	GCA_013418535.1
323	YH12140	tet(X3)	Acinetobacter amyesii	-	2017	China	pig	GCA_013418475.1
324	YH12141	tet(X3)	Acinetobacter variabilis	-	2017	China	pig	GCA_013418465.1
325	YH12142	tet(X3)	Acinetobacter amyesii	-	2017	China	pig	GCA_013418435.1
326	YH12143	tet(X3)	Acinetobacter variabilis	-	2017	China	sewage	GCA_013418375.1
327	YH12144	tet(X3)	Acinetobacter amyesii	-	2017	China	pig	GCA_013418345.1
328	YH12145	tet(X3)	Acinetobacter amyesii	-	2017	China	pig	GCA_013418335.1
329	YH12151	tet(X3)	Acinetobacter pseudolwoffii	-	2017	China	pig	GCA_013418355.1
330	YH12152	tet(X3)	Acinetobacter amyesii	-	2017	China	pig	GCA_013418285.1
331	YH12153	tet(X3)	Acinetobacter pseudolwoffii	-	2017	China	pig	GCA_013418275.1
332	YH12154	tet(X3)	Acinetobacter amyesii	-	2017	China	pig	GCA_013418265.1
333	YH12155	tet(X3)	Acinetobacter indicus	-	2017	China	pig	GCA_013418255.1
334	YH12156	tet(X3)	Acinetobacter pseudolwoffii	-	2017	China	pig	GCA_013418235.1
335	YH12157	tet(X3)	Acinetobacter indicus	-	2017	China	pig	GCA_013418215.1
336	YH12205	tet(X3)	Acinetobacter towneri	-	2017	China	pig	GCA_013418125.1
337	YH12208	tet(X3)	Acinetobacter pseudolwoffii	-	2017	China	soil	GCA_013418055.1
338	YH12211	tet(X3)	Acinetobacter towneri	-	2017	China	pig	GCA_013418015.1
339	YH12212	tet(X3)	Acinetobacter indicus	-	2017	China	pig	GCA_013417955.1
340	YH12214	tet(X3)	Acinetobacter indicus	-	2017	China	pig	GCA_013417945.1
341	YH12216	tet(X3)	Acinetobacter indicus	-	2017	China	pig	GCA_013417935.1
342	YH12217	tet(X3)	Acinetobacter indicus	-	2017	China	pig	GCA_013417915.1
343	YH12218	tet(X3)	Acinetobacter amyesii	-	2017	China	pig	GCA_013417925.1
344	YH12222	tet(X3)	Acinetobacter indicus	-	2017	China	pig	GCA_013417885.1
345	YH12223	tet(X3)	Acinetobacter indicus	-	2017	China	pig	GCA_013417855.1
346	YH12224	tet(X3)	Acinetobacter indicus	-	2017	China	pig	GCA_013417795.1

347	YH12226	tet(X3)	Acinetobacter lwoffii	-	2017	China	vegetable	GCA_013417805.1
348	YH12227	tet(X3)	Acinetobacter gandensis	-	2017	China	vegetable	GCA_013417785.1
349	YH12230	tet(X3)	Acinetobacter indicus	-	2015	China	pig	GCA_013417775.1
350	YH12231	tet(X3)	Acinetobacter amyesii	-	2015	China	pig	GCA_013417755.1
351	YH12233	tet(X3)	Acinetobacter variabilis	-	2016	China	pig	GCA_013417725.1
352	YH12236	tet(X3)	Acinetobacter variabilis	-	2016	China	pig	GCA_013417645.1
353	YH12237	tet(X3)	Acinetobacter towneri	-	2016	China	pig	GCA_013417665.1
354	YH12238	tet(X3)	Acinetobacter towneri	-	2016	China	pig	GCA_013417655.1
355	YH12239	tet(X3)	Taxon 58	-	2016	China	pig	GCA_013417635.1
356	YH12241	tet(X3)	Acinetobacter variabilis	-	2016	China	pig	GCA_013417535.1
357	YH12242	tet(X3)	Acinetobacter variabilis	-	2016	China	pig	GCA_013417555.1
358	YH12243	tet(X3)	Acinetobacter variabilis	-	2016	China	pig	GCA_013417545.1
359	YH12244	tet(X3)	Acinetobacter variabilis	-	2016	China	pig	GCA_013417565.1
360	YH12245	tet(X3)	Acinetobacter variabilis	-	2016	China	pig	GCA_013417575.1
361	YH12247	tet(X3)	Acinetobacter variabilis	-	2016	China	pig	GCA_013417485.1
362	YH12248	tet(X3)	Acinetobacter variabilis	-	2016	China	pig	GCA_013417455.1
363	YH12249	tet(X3)	Acinetobacter variabilis	-	2016	China	dust	GCA_013417475.1
364	YH12251	tet(X3)	Acinetobacter amyesii	-	2017	China	pig	GCA_013417465.1
365	YH12252	tet(X3)	Acinetobacter towneri	-	2017	China	pig	GCA_013417365.1
366	YH12254	tet(X3)	Acinetobacter pseudolwoffii	-	2017	China	pig	GCA_013417415.1
367	YH12255	tet(X3)	Acinetobacter pseudolwoffii	-	2017	China	pig	GCA_013417355.1
368	YH16031	tet(X3)	Acinetobacter variabilis	-	2016	China	soil	GCA_013417335.1
369	YH16037	tet(X3)	Acinetobacter amyesii	-	2016	China	pig	GCA_013417265.1
370	YH16040	tet(X3)	Acinetobacter amyesii	-	2016	China	pig	GCA_013417235.1
371	YH16042	tet(X3)	Acinetobacter amyesii	-	2016	China	pig	GCA_013417245.1

372	YH16044	tet(X3)	Acinetobacter amyesii	-	2016	China	pig	GCA_013417145.1
373	YH16050	tet(X3)	Acinetobacter towneri	-	2016	China	sewage	GCA_013417165.1
374	YH16053	tet(X3)	Acinetobacter towneri	-	2016	China	pig	GCA_013417035.1
375	YH16055	tet(X3)	Acinetobacter amyesii	-	2016	China	pig	GCA_013417045.1
376	YH16056	tet(X3)	Acinetobacter amyesii	-	2016	China	soil	GCA_013417055.1
377	YH16057	tet(X3)	Acinetobacter amyesii	-	2016	China	pig	GCA_013417075.1
378	YH16058	tet(X3)	Acinetobacter towneri	-	2016	China	pig	GCA_013417065.1
379	YH18001	tet(X3)	Acinetobacter pseudolwoffii	-	2017	China	homo sapiens	GCA_013416975.1
380	YH1901130	tet(X3)	Acinetobacter amyesii	-	2017	China	pig	GCA_013416955.1
381	YH1901136	tet(X3)	Acinetobacter towneri	-	2016	China	pig	GCA_013416945.1
382	YH1901141	tet(X3)	Acinetobacter towneri	-	2017	China	pig	GCA_013416935.1
383	YH1901147	tet(X3)	Acinetobacter towneri	-	2017	China	soil	GCA_013416875.1
384	YH1901152	tet(X3)	Acinetobacter variabilis	-	2017	China	pig	GCA_013416895.1
385	ZZC3-9	tet(X3)	Taxon 91	-	2018	China	cattle	GCA_015351655.1
386	CRAb2	tet(X3)	Acinetobacter baumannii	32	2021	Netherlands	homo sapiens	GCA_038441325.1
387	CRAb1	tet(X3)	Acinetobacter baumannii	32	2021	Netherlands	homo sapiens	GCA_038442095.1
388	AIBD14	tet(X6)	Acinetobacter indicus	-	2022	China	slaughterhouse waste	GCA_041930565.1
389	PT23-B2	tet(X7)	Acinetobacter towneri	-	2023	Philippines	hospital wastewater	GCA_040436385.1
390	CCRI-1017	tet(X3)	Acinetobacter baumannii	109	-	Argentina	homo sapiens	GCA_045066655.1

[a] -, not collected.
